# Intraarticular triamcinolone hexacetonide, stanozolol, Hylan G-F 20 and platelet concentrate in a naturally occurring canine osteoarthritis model

**DOI:** 10.1038/s41598-021-82795-z

**Published:** 2021-02-04

**Authors:** J. C. Alves, A. Santos, P. Jorge, C. Lavrador, L. Miguel Carreira

**Affiliations:** 1 Divisão de Medicina Veterinária, Guarda Nacional Republicana (GNR), Rua Presidente Arriaga, 9, 1200-771 Lisbon, Portugal; 2 MED – Mediterranean Institute for Agriculture, Environment and Development, Instituto de Investigação e Formação Avançada, Universidade de Évora, Pólo da Mitra, Ap. 94, 7006-554 Évora, Portugal; 3 Faculty of Veterinary Medicine, University of Lisbon (FMV/ULisboa), Lisbon, Portugal; 4 Interdisciplinary Centre for Research in Animal Health (CIISA) – University of Lisbon, (FMV/ULisboa), Lisbon, Portugal; 5 Anjos of Assis Veterinary Medicine Centre (CMVAA), Barreiro, Portugal

**Keywords:** Osteoarthritis, Cartilage

## Abstract

Osteoarthritis (OA) is a disease transversal to all mammals, a source of chronic pain and disability, a huge burden to societies, with a significant toll in healthcare cost, while reducing productivity and quality of life. The dog is considered a useful model for the translational study of the disease, closely matching human OA, with the advantage of a faster disease progression while maintaining the same life stages. In a prospective, longitudinal, double-blinded, negative controlled study, one hundred (N = 100) hip joints were selected and randomly assigned to five groups: control group (CG, n = 20, receiving a saline injection), triamcinolone hexacetonide group (THG, n = 20), platelet concentrate group (PCG, n = 20), stanozolol group (SG, n = 20) and hylan G-F 20 group (HG). Evaluations were conducted on days 0 (T0, treatment day), 8, 15, 30, 60, 90, 120, 150 and 180 days post-treatment, consisting of weight distribution analysis and data from four Clinical Metrology Instruments (CMI). Kaplan–Meier estimators were generated and compared with the Breslow test. Cox proportional hazard regression analysis was used to investigate the influence of variables of interest on treatment survival. All results were analyzed with IBM SPSS Statistics version 20 and a significance level of p < 0.05 was set. Sample included joints of 100 pelvic limbs (of patients with a mean age of 6.5 ± 2.4 years and body weight of 26.7 ± 5.2 kg. Joints were graded as mild (n = 70), moderate (n = 20) and severe (n = 10) OA. No differences were found between groups at T0. Kaplan–Meier analysis showed that all treatments produced longer periods with better results in the various evaluations compared to CG. Patients in HG and PCG took longer to return to baseline values and scores. A higher impact on pain interference was observed in THG, with a 95% improvement over CG. PCG and HG experienced 57–81% improvements in functional evaluation and impairments due to OA, and may be a better options for these cases. This study documented the efficacy of several approaches to relieve OA clinical signs. These approaches varied in intensity and duration. HG and PCG where the groups were more significant improvements were observed throughout the follow-up periods, with lower variation in results.

## Introduction

Osteoarthritis (OA) is a disease transversal to all mammals^1^. Being a source of chronic pain and disability, it represents a huge burden to societies, with a significant toll in healthcare cost, while reducing productivity and quality of life^2,3^. Its prevalence is expected to rise, due to a simultaneous increase in life expectancy and obesity^4^. The pathologic process, clinical presentation and response to treatment are very similar in humans and dogs, making the dog a frequent animal model for the study of osteoarthritis^5^. In fact, the changes that occur in slowly progressive spontaneous dog OA closely match those of human OA, with the added advantage of a faster disease progression while maintaining a juvenile, adolescent, adult and geriatric life stages. In addition, companion animals share many of the same environmental conditions that their human counterparts. For those reasons the natural occurring canine model is considered an useful model of human OA, and exploring spontaneous canine OA can help improve human and dog health^6–11^.

The medical approach to OA aims at slowing disease progression while relieving symptoms, particularly pain, and improving overall function^9,12^. Imaging plays a key role in the assessment of patients with joint disease. In the case of hip OA, the ventrodorsal (VD) hip extended view is the most commonly performed radiographic view^13,14^. This view is a valuable tool for evaluating the presence of hip OA^15^. Affected patients commonly bear less weight on an affected limb, since OA pain is related to movement or weight-bearing impairments of the affected joints. Evaluating weight distribution through stance analysis is a sensitive evaluation of lameness in dogs^16–19^. Weight distribution and off-loading or limb favouring at the stance are commonly used subjective assessments during the orthopaedic examination^20^. Dogs with OA may not be overtly lame at a walk or a trot but exhibit subtle shifts in body weight distribution at a stance due to pain^18,21^. Stance analysis has been reported as sensitive for detecting lameness in dogs, proposed to be an equivalent or superior measurement of pain associated with hip OA than vertical impulse and peak vertical force VI and PVF^18^. OA pain is a multi-dimensional experience, which encompasses more than just a functional aspect, and treatment interventions must address this reality^17,22,23^. Clinical metrology instruments (CMI) represent a patient-centred approach, and the most commonly used ones to evaluate dogs are the Canine Brief Pain Inventory (CBPI, divided in a pain severity score—PSS, and a pain interference score—PIS) and the Liverpool Osteoarthritis in Dogs (LOAD). In addition, the Hudson Visual Analogue Scale (HVAS), developed to assess the degree of lameness in dogs, and the Canine Orthopaedic Index (COI, divided in four scores: stiffness, gait, function and quality of life—QOL) are further validated CMIs which can complement the evaluation of the multi-dimensional, not directly measured experience that is OA related pain^9,17,19,24–30^.

IA therapies several advantages over systemic medications, as safety, especially when certain comorbidities are present, and bioavailability^31^. IA corticosteroids have been used for several decades to palliate pain and inflammation associated with OA and surrounding tissues^32,33^. Triamcinolone hexacetonide (TH), in particular, is described as able to provide pain relief and improved mobility for prolonged periods^34,35^. Autologous platelets are a regenerative treatment modality for OA, used with the aim to stimulate the natural healing cascade and regeneration of tissues, through a supraphysiologic release of growth factors directly at the treatment site^36–39^. Stanozolol is a synthetic derivative of testosterone, and its properties include anabolic/androgenic activity^40^. When administered IA, it is able to induce fibroblasts to increase collagen production, decrease nitric oxide production and induce osteoblast proliferation and collagen synthesis^41–44^. It also has a chondroprotective and cartilage regeneration effect, while reducing osteophyte formation and subchondral bone reaction^42,45^. Even though hyaluronan’s mechanism of action is not completely known and clinical trials have provided contradictory results, the aim of its use in the treatment of patients with OA is to reduce pain and improve function by supplementing the viscosity and elasticity of synovial fluid^46,47^. Additional anti-inflammatory, anti-nociceptive and chondroprotective properties have been suggested^48,49^. High molecular weight products seem to produce better results, particularly in patients with mild radiographic disease^50,51^.

In order to assess long-term outcomes and to identify factors associated with poorer outcome, we compared the effect of the intraarticular administration triamcinolone hexacetonide, hylan G-F 20, stanozolol and a platelet concentrate in the management of OA in a natural occurring canine model. We hypothesize that the different treatments will be able to reduce the clinical signs of OA, compared to a control group.

## Results

The sample included 100 pelvic limbs (n = 50 left and n = 50 right) of fifty active Police working dogs, with a mean age of 6.5 ± 2.4 years and body weight of 26.7 ± 5.2 kg, representing both sexes (male n = 60, female n = 40). They were housed in kennels of the Portuguese Gendarmerie Canine Unit, similar in size. All dogs remained in active work during and after the study, and engaged in search and rescue, product detection and use of force mission. Active work and training were conducted on a daily basis, with their individual handlers. At T0, 70 joints were classified as mild, 20 as moderate and 10 as severe, according to the Orthopedic Foundation for Animals hip grading scheme. Values and scores of each evaluation in all groups at T0 are presented in Table 1. No differences were found between groups at the initial evaluation (p = 0.22 for SI, p = 0.075 for deviation, p = 0.12 for HVAS, p = 0.23 for PSS, p = 0.22 for PIS, p = 0.07 for LOAD, p = 0.48 for stiffness, p = 0.10 for function, p = 0.46 for gait, p = 0.25 for QOL and p = 0.21 for COI).

**Table 1:** Mean values (± standard deviation) at initial evaluation of evaluation conducted for control and treatment groups.

	CG	THG	HG	SG	PCG
Weight (kg. mean ± SD)	28.2 ± 6.2	26.5 ± 6.5	26.7 ± 3.5	27.1 ± 3.2	25.1 ± 9.6
Deviation (mean ± SD)	3.6 ± 2.7	4.7 ± 4.4	3.8 ± 3.5	4.3 ± 3.5	4.1 ± 2.2
Symmetry Index (mean ± SD)	24.8 ± 26.5	23.9 ± 50.4	21.7 ± 24.9	24.1 ± 13.9	22.6 ± 12.4
HVAS (0–10)	6.8 ± 1.2	5.7 ± 1.9	6.6 ± 1.4	6.7 ± 1.3	6.7 ± 1.4
PSS (0–10)	3.1 ± 1.9	4.2 ± 2.8	3.3 ± 2.6	2.9 ± 1.5	3.3 ± 2.6
PIS (0–10)	2.8 ± 1.7	4.8 ± 3.3	3.4 ± 2.3	2.3 ± 1.7	3.3 ± 2.8
LOAD (0–52)	13.6 ± 10.5	23.2 ± 14.1	17.0 ± 10.5	8.2 ± 5.2	13.3 ± 11.3
Stiffness (0–16)	3.4 ± 3.4	6.8 ± 4.2	3.4 ± 2.9	4.0 ± 2.8	3.9 ± 3.9
Function (0–16)	3.6 ± 4.1	6.3 ± 5.7	4.6 ± 3.5	4.0 ± 3.6	4.2 ± 4.7
Gait (0–20)	5.3 ± 3.9	10.5 ± 5.9	7.4 ± 4.7	5.2 ± 3.9	5.1 ± 5.4
QOL (0–12)	4.3 ± 2.5	6.2 ± 3.9	4.5 ± 3.1	4.3 ± 2.5	4.5 ± 3.6
COI (0–64)	17.6 ± 12.4	19.8 ± 19.1	19.9 ± 127	17.5 ± 12.4	17.7 ± 16.9

*CG* Control group, *THG* Triamcinolone hexacetonide group, *HG* Hylan G-F 20 group, *SG* Stanozolol group, *PCG* Platelet concentrate group, *HVAS* Hudson Visual Analogue Scale, *PSS* Pain Severity Score, *PIS* Pain Interference Score, *LOAD* Liverpool Osteoarthritis in Dogs, *QOL* Quality of Life, *COI* Canine Orthopedic Index.

All patients were followed up to the last evaluation moment (180 days) and, during this period, no additional treatment or medications was administered. Results of the Kaplan–Meier estimators with each evaluation method are presented in Table 2. All treatments showed longer periods with better results in the various evaluations compared to CG. Patients in HG and PCG, in particular, took longer to return to baseline values and scores. Results of the Cox proportional hazard regression are presented in Table 3. Increased lameness was observed in 16 patients in PCG, 8 in SG, 6 in HG and 4 in THG, which spontaneously resolved within 48–72 h.

**Table 2:** Survival probability calculated with Kaplan–Meier estimators and compared with the Breslow test.

Variable	Breslow test	Treatment									
		CG		THG		SG		HG		PCG	
		Mean ± SD	95% CI	Mean ± SD	95% CI	Mean ± SD	95% CI	Mean ± SD	95% CI	Mean ± SD	95% CI
Symmetry Index	0.000*	47.0 ± 11.8	23.8–70.2	96.0 ± 12.8	70.9–121.1	94.2 ± 15.9	62.9–125.4	104.1 ± 15.1	15.1–74.5	159.0 ± 10.3	138.9–179.1
Deviation	0.000*	44.8 ± 12.1	21.1–68.5	81.8 ± 14.7	52.9–110.6	55.6 ± 11.8	32.3–78.8	96.2 ± 16.3	64.2–128.1	138.0 ± 12.5	113.4–162.6
HVAS	0.000*	48.7 ± 12.4	25.4–73.9	66.1 ± 14.2	38.3–93.9	129.8 ± 13.4	103.5–156.1	117.0 ± 13.2	91.1–142.9	144.0 ± 11.6	121.2–166.8
PSS	0.015*	63.2 ± 17.2	29.6–96.8	90.2 ± 17.6	55.7–124.7	94.6 ± 16.4	62.5–126.7	142.6 ± 11.9	119.1–166.0	150.5 ± 9.7	130.9–169.1
PIS	0.000*	8.4 ± 0.4	7.7–9.0	118.6 ± 16.3	86.7–150.5	109.6 ± 17.3	75.8–143.2	114.0 ± 16.0	82.6–145.4	135.0 ± 10.6	114.2–155.8
LOAD	0.000*	40.7 ± 10.6	19.9–61.4	124.3 ± 15.9	93.1–155.5	123.8 ± 14.2	95.9–151.6	141.8 ± 11.6	119.2–164.4	120.0 ± 12.8	94.9–145.0
Stiffness	0.009*	64.7 ± 16.9	31.4–97.9	130.8 ± 11.6	108.1–153.5	111.2 ± 15.9	80.6–142.9	129.8 ± 13.9	102.6–157.0	141.0 ± 16.9	119.9–162.1
Function	0.000*	65.4 ± 13.4	39.2–91.6	112.6 ± 15.6	81.9–143.2	124.5 ± 15.4	94.2–154.8	168.0 ± 6.6	155.1–180.8	135.0 ± 9.4	116.5–153.5
Gait	0.001*	52.7 ± 14.6	23.9–81.4	117.0 ± 15.1	87.5–146.5	103.6 ± 15.7	72.8–134.4	115.5 ± 13.1	89.9–141.1	123.0 ± 12.5	98.5–147.5
QOL	0.004*	60.9 ± 15.0	31.4–90.4	119.3 ± 17.5	85.0–153.6	66.2 ± 17.5	31.8–100.6	125.6 ± 12.2	101.6–149.6	120.8 ± 13.1	95.1–146.5
COI	0.011*	52.7 ± 13.4	26.5–78.9	85.6 ± 15.9	54.4–116.9	78.1 ± 14.0	50.6–105.6	93.1 ± 16.7	60.3–125.9	138.0 ± 10.8	116.9–159.1

See Table 1 for legend.

*Indicates significance.

**Table 3:** Results Cox proportional hazard regression with the different outcome evaluations.

Variable	Wheight distribution				HVAS (p = 0.000)		CBPI				LOAD (p = 0.000)	
	Symmetry Index (p = 0.008)		Deviation (p = 0.024)				PSS (p = 0.412)		PIS (p = 0.000)			
	HR (95% CI)	p	HR (95% CI)	p	HR (95% CI)	p	HR (95% CI)	p	HR (95% CI)	p	HR (95% CI)	p
Age	0.91 (0.83–10.01)	0.074	0.95 (0.86–1.05)	0.314	1.13 (1.02–1.25)	0.024*	1.08 (0.97–1.21)	0.163	0.95 (0.86–1.06)	0.374	0.93 (0.83–1.04)	0.190
Body weight			1.02 (0.97–1.07)	0.509	0.98 (0.93–1.03)	0.378	0.99 (0.93–1.05)	0.770	1.02 (0.97–1.08)	0.479	0.99 (0.94–1.06)	0.941
**Sex**												
Male	1.00				1.00		1.00		1.00		1.00	
Female	1.09 (0.59–2.02)	0.763	0.88 (0.49–1.57)	0.657	0.31 (0.15–0.62)	0.001*	1.24 (0.64–2.38)	0.524	1.12 (0.61–2.08)	0.715	1.18 (0.63–2.21)	0.616
Treatment		0.002*		0.005*		0.003*		0.154		0.000*		0.000*
Control	1.00				1.00		1.00		1.00		1.00	
HG	0.53 (0.27–1.04)	0.067	0.44 (0.22–0.88)	0.020*	0.27 (0.12–0.63)	0.002*	0.31 (0.12–0.78)	0.013*	0.07 (0.03–0.21)	0.000*	0.11 (0.04–0.28)	0.000*
PCG	0.20 (0.93–0.45)	0.000*	0.31 (0.15–0.62)	0.001*	0.26 (0.12–0.56)	0.001*	0.53 (0.24–1.16)	0.114	0.09 (0.03–0.24)	0.000*	0.31 (0.15–0.64)	0.001*
SG	0.39 (0.19–0.82)	0.012*	0.85 (0.44–1.66)	0.642	0.33 (0.14–0.77)	0.010*	0.57 (0.25–1.33)	0.195	0.08 (0.03–0.23)	0.000*	0.17 (0.07–0.37)	0.000*
THG	0.46 (0.22–0.96)	0.039*	0.43 (0.21–0.89)	0.023*	0.98 (0.93–1.03)	0.151	0.70 (0.31–1.59)	0.397	0.05 (0.02–0.14)	0.000*	0.09 (0.94–1.06)	0.000*
OFA score		0.831		0.179		0.026*				0.175		0.007*
Mild	1.00				1.00		1.00		1.00		1.00	
Moderate	0.85 (0.45–1.58)	0.605	1.01 (0.57–1.80)	0.973	1.29 (0.68–2.47)	0.435	1.09 (0.58–2.03)	0.79	1.74 (0.95–3.19)	0.074	2.93 (1.50–5.70)	0.002*
Severe	1.11 (0.47–2.62)	0.811	2.21 (0.95–5.2)	0.67	3.46 (1.39–8.55)	0.007*	0.79 (0.25–2.5)	0.70	0.86 (0.29–2.5)	0.784	1.56 (0.49–4.92)	0.451

Variable	COI											
	Stiffness (p = 0.001)		Function (p = 0.000)		Gait (p = 0.000)		QOL (p = 0.033)		Total (p = 0.000)			
	HR (95% CI)	p	HR (95% CI)	p	HR (95% CI)	P	HR (95% CI)	p	HR (95% CI)	p		
Age	1.19 (1.05–1.35)	0.007*	1.09 (0.99–1.21)	0.093	1.27 (1.13–1.43)	0.000*	0.98 (0.88–0.11)	0.835	1.15 (1.04–1.26)	0.005*		
Body weight	1.03 (0.97–1.09)	0.324	1.03 (0.97–1.09)	0.346	1.01 (0.96–1.07)	0.717	1.04 (0.99–1.10)	0.106	0.98 (0.93–1.05)	0.603		
**Sex**												
Male	1.00		1.00		1.00		1.00		1.00			
Female	2.02 (0.99–4.09)	0.051	1.40 (0.74–2.67)	0.304	1.28 (0.67–2.45)	0.453	2.58 (1.33–5.01)	0.005*	1.79 (0.95–3.37)	0.071		
Treatment		0.032*		0.000*		0.002*		0.303		0.012*		
Control	1.00		1.00		1.00		1.00		1.00			
HG	0.25 (0.09–0.64)	0.004*	0.09 (0.03–0.27)	0.000*	0.16 (0.06–0.42)	0.000*	0.45 (0.19–1.05)	0.064	0.41 (0.19–0.90)	0.026*		
PCG	0.34 (0.15–0.79)	0.012*	0.40 (0.19–0.81)	0.011*	0.28 (0.13–0.61)	0.001*	0.55 (0.26–1.15)	0.113	0.28 (0.13–0.59)	0.001*		
SG	0.41 (0.16–1.02)	0.054	0.27 (0.11–0.66)	0.004*	0.45 (0.19–1.03)	0.058	0.92 (0.45–1.85)	0.808	0.53 (0.24–1.14)	0.103		
THG	0.34 (0.14–0.80)	0.014*	0.33 (0.15–0.73)	0.006*	0.32 (0.15–0.69)	0.004*	0.69 (0.32–1.47)	0.333	0.39 (0.18–0.85)	0.018*		
OFA score		0.0159		0.014*		0.081		0.338		0.048*		
Mild	1.00		1.00		1.00		1.00		1.00			
Moderate	0.97 (0.49–1.89)	0.936	1.07 (0.54–2.14)	0.841	0.66 (0.34–1.28)	0.222	0.81 (0.44–1.47)	0.477	0.61 (0.33–1.15)	0.127		
Severe	2.48 (0.94–6.59)	0.068	4.59 (1.65–12.82)	0.004*	2.11 (0.79–5.58)	0.134	1.75 (0.67–4.55)	0.252	2.17 (0.86–5.45)	0.101		

See Table 1 for legend.

*Indicates significance.

## Discussion

Osteoarthritis is a leading cause of disability around the world, which affects both the physical and mental well-being of populations. It poses a huge toll on healthcare resources and productivity^52^. Despite extensive research, still has limited treatment options available^9,12^. To our knowledge, this is the first prospective, negative controlled, double-blinded study to compare the effect of commonly used and novel IA treatments for the management of OA, in a naturally occurring canine model, with a long follow up period.

Human reports on the effect of IA TH describe its long term safety, with improvements in joint range of motion and pain compared with a saline injection, with no differences between treatment with 40 mg or 20 mg of TH^53–55^. Previous reports on the effect of a single administration of a platelet concentrate showed improvements in pain, kinetics and joint range of motion, up to the last evaluation point considered, which ranged from 12 weeks to 6 months^56,57^. Different reports on the use of stanozolol in animals described it as being able to resolve signs of lameness, while reducing osteophyte formation, subchondral bone reaction, and promoting articular cartilage regeneration^42,43^. A previous study on a canine model has provided information on the efficacy of IA hyaluronan in animals with OA of pain, function, lameness and kinetics when compared to pre-treatment and saline control, with maximum benefits noted at 4–8 weeks and gradually tampered down by a 6-month evaluation time point^58^. OA is characterized by variable degrees of clinical and functional impairments, with the severity of pain correlating with the functional status rather than radiographic grading of osteoarthritis, which does not correlate with functional status. Treatment, therefore, could be planned according to the clinical features and functional status instead of radiological findings^59,60^. For that reason, we compared well established to novel therapeutic approaches, while evaluating the impact of documented predisposing and clinical factors of OA. All treatments were able to improve clinical signs of OA compared with the treatment groups, in all of the evaluated dimensions, from pain, function and quality of life. As a whole, patients in PCG and HG took longer to return to baseline values, which may indicate that these approaches are better therapeutic approaches for the management of OA. It was interesting to see that patients in the CG did not remained or returned to initial values and scores at the first follow-up evaluations, in some cases taking 60 days to do so. It has been documented that placebo saline injections may have an effect in functional improvements that can last up to a 6-month follow-up^61^, and a similar phenomenon may have occurred in this study.

We also investigated variables which could influence patient’s response to treatment, regarding different OA dimensions. Age showed an impact in functional scores as HVAS, stiffness, gait and COI, as did the degree of OA, with patients with severe OA showing an impact on HVAS and function score. These findings could be explained with the fact that OA is a progressive, degenerative disease, which ultimately has a toll on joint function, without, however, a corresponding increase in pain levels. It is also well established that individuals with higher body mass index experience greater pain than individuals with lower index^62^. We evaluated the effect of body weight in the evolution of OA, which is not the same as body mass index, but since patients in this sample were working dogs, with an ideal body condition score, we chose to evaluate body weight instead which did not influenced any of the evaluation made.

Comparing the effect of different treatments, as a whole they had an effect on all dimensions evaluated, except PSS. Reasons for that may be related with the nature of these animals are working dogs and, for that reason, tend to show few signs of overt pain. In fact, pain is more easily and commonly detected through its impact on measurable parameters, such as weigh bearing, or on active exercises^57^. This may be reflected on an effect of different treatments on the PIS score, but not on the PSS score. PCG and HG registered effects for longer periods, and better improvements according to the Cox hazard regression with the different evaluations made. Considering measurable parameters, patients in PCH showed an 81% and 69% improvement in SI and deviation, respectively, while HG showed 61% and 57% improvements. These seem to be the preferred treatments for functional impairments due to OA. In addition to these evaluations, PCG and HG also registered greater improvements in several scores as HVAS, stiffness, function, gait and COI. Better impact on pain interference was observed in THG, which could be attributed to the high anti-inflammatory effect of corticosteroids, and the relation between pain and inflammation.

Side effects related after IA treatment are documented, and usually include injection pain and local inflammation, that take 2–10 days to resolve^43,58,63,64^. We observed increased lameness in all groups, which spontaneously resolved within 48–72 h.

This study presents some limitations, namely the inclusion of two joints from each dogs, as an association may occur between limbs. This effect has been described in humans^65,66^. Still, the inclusion of contralateral limbs from the same patient is common in animal models, even for the calculation of a symmetry index^27,42,58^. A reason for this is that quadrupeds show more complex compensation mechanism than just side-to-side. Results from the weight bearing evaluation of the patients of this study show that the major compensation occurs in the contralateral thoracic limb, rather than side-to-side. In addition, a majority of joints considered in this study had mild OA. Further studies should include a larger number of the remaining hip grades to determine if similar results are obtained. The safety and efficacy of repeated IA injections should also be investigated.

## Conclusions

To our knowledge, this is the first prospective, negative controlled, double-blinded study to compare the effect of commonly used and novel IA treatments for the management of OA, in a naturally occurring canine model, with a long follow up period. It provides important information for the characterization of the effects of these treatment modalities, duration of observed improvements function and pain, in addition to information regarding candidates for each one.

## Methods

The study protocol was approved by the ethical review committee of the University of Évora (Órgão Responsável pelo Bem-estar dos Animais da Universidade de Évora, approval nº GD/32055/2018/P1, September 25th, 2018), and complies with ARRIVE guidelines. All experiments were performed in accordance with relevant guidelines and regulations. Written, informed consent was obtained from the Institution responsible for the animals. In a prospective, longitudinal, double-blinded, negative controlled study, patients were selected after screening of the Portuguese Gendarmerie Canine Unit, based on history, physical, orthopedic, neurological and radiographic examinations compatible with bilateral hip OA. The sample comprised one hundred (N = 100) hip joints of fifty active Police working dogs. It constituted a convenience sample, similar in size to previously published reports on this topic^58,67,68^. Inclusion criteria comprised age over two years, bodyweight over 20 kg, symptomatic in both limbs, with the same OA grade on both hips, and patients should not have received any medication or nutritional supplement for over six weeks. Cases with any other documented or suspected orthopaedic or neurological disease, or any other concomitant disease, were ruled out through physical and radiographic, examination, complete blood count and serum chemistry profile.

After selection, patients were randomly assigned to one of five groups, 10 dogs per group, and treated bilaterally: control group (CG, n = 20 joints), triamcinolone hexacetonide group (THG, n = 20 joints), platelet concentrate group (PCG, n = 20 joints), stanozolol group (SG, n = 20 joints) and hylan G-F 20 group (HG, n = 20 joints). Evaluations were conducted on days 0 (treatment day), 8, 15, 30, 60, 90, 120, 150 and 180 days post treatment. An outline of all procedures on each moment is presented in Table 4. All evaluations and procedures were performed by the same researcher.

**Table 4:** Procedures conducted in each evaluation moment. Days are counted from treatment day.

Procedure	Day								
	0	8	15	30	60	90	120	150	180
Treatment	X								
Stance analysis	X	X	X	X		X			X
Digital radiography	X			X		X			X
HVAS	X	X	X	X	X	X	X	X	X
CBPI	X	X	X	X	X	X	X	X	X
COI	X	X	X	X	X	X	X	X	X
LOAD	X	X	X	X	X	X	X	X	X

*CBPI* Canine Brief Pain Inventory, *COI* Canine Orthopedic Index, *HVAS* Hudson Visual Analogue Scale, *LOAD* Liverpool Osteoarthritis in Dogs.

On treatment day, patients in CG received an IA administration of 2 ml of 0.9%NaCl, given IA. On the same day, patients in THG received an IA administration of 20 mg/1 ml of triamcinolone hexacetonide (Bluxam, Riemser Pharma, Portugal). In SG, IA administration of stanozolol (Estrombol, Laboratório Fundacion), at a 0.3 mg/kg dose was performed^69,70^. Patients in HG received 2 ml of hylan G-F 20 (Synvisc, Sanofi, Portugal). For patients in PCG, 3 ml of platelet concentrate, prepared with the commercially available V-PET kit, according to the manufacturer’s instructions, was applied. For the preparation of the platelet concentrate, fifty-five milliliters of whole blood were collected from the jugular vein of the patient and then introduced into the provided closed system. There, the blood flowed by action of gravity through a filter, where the platelets where concentrated. The final platelet concentrate was then collected and was used in the following 5 min of its preparation.

IA administrations and radiographic examination were conducted under light sedation, induced with a combination of medetomidine (0.01 mg/kg) and buthorphanol (0.1 mg/kg), given intravenously. A VD extended legs projection was used, and joints classified according to the Orthopedic Foundation for Animals hip grading scheme^14^. A full description of the OFA hip grading scheme is available online. For the IA administration, patients were placed in lateral recumbency, with the affected limb uppermost. A window of 4 × 4 cm in the area surrounding the greater trochanter was clipped and aseptically prepared. The limb was then placed in a neutral position, parallel to the table. A 21-gauge with 2.5″ length needle was then introduced just dorsal to the greater trochanter, perpendicular to the long axis of the limb, until the joint was reached^71^. Confirmation of correct needle placement was obtained through the collection of synovial fluid, and the treatment or saline were administered. Stance analysis was conducted with a weight distribution platform (Companion Stance Analyzer; LiteCure LLC, Newark, Delaware, United States). The equipment was placed in the centre of an observation room, at least 1-m from the walls. Complying with manufacturer’s guidelines, the platform was calibrated at the beginning of each testing day and zeroed before each data collection. After an acclimatization period, animals were encouraged to stand on the weight distribution platform. To secure a correct position, the patient’s trainer helped to ensure it placed one foot on each quadrant of the platform, while maintaining a natural stance with its their centre of gravity and stability (measured by the platform) near the middle of the platform. When required, gentle restraint was used to maintain the patient’s head in a natural, forward-facing position. For all animals, at least 20 measurements were performed, and the mean value was determined. The left–right symmetry index (SI) was calculated according to the following formula: SI = [(WB_R_ − WB_L_)/((WB_R_ + WB_L_) × 0.5)] × 100^27,72^, where WB_R_ is the value of weight-bearing for the right pelvic limb and WB_L_ is the value of weight-bearing for the left pelvic limb. Negative values were made positive. We also considered deviation from the normal 20% weight-bearing for a pelvic limb^18^, calculated by subtracting WB to 20_._ Before completion of an online copy of the HVAS, CBPI, COI and LOAD, handlers received the published instructions for each of them. The CMIs were completed sequentially by the same handler in each of the follow-up assessments, without knowledge of their previous answers, in a calm room with as much time as needed to answer all items. As CBPI has two section (PSS and PIS), and COI has four dimensions (stiffness, function, gait and QOL), were considered all sections and dimensions in the analysis. After treatment, animals were rested for three consecutive days and resumed their normal activity over a period of 5 days. On days 1 and 3 after the procedure, the veterinarian examined all patients in order to determine possible signs of exacerbated pain, persistent stiffness of gait and changes in posture. If no complaints were registered, the animal could resume its normal activity^73,74^. If a deterioration of the animal’s condition was detected, rescue analgesia would be provided and based on the administration of a combination of opioids (tramadol, 2–5 mg/kg BID or TID) and gabapentin (10–20 mg/kg TID), as needed.

The outcome considered was as return to or drop below baseline values of SI or deviation and scores of the considered CMIs at the 180-day post treatment. Demographic data consisting of age, sex and body weight, was noted. Results are expressed as mean ± SD. Kaplan–Meier estimators were conducted to generate survival curves, survival probability and compared with the Breslow test. Cox proportional hazard regression analysis was carried out to investigate the influence of the variables of interest (age, sex, body weight and OFA score) on treatment survival. Treatments were compared to control at initial evaluation with a Wilcoxon signed-rank test. Patients with values or scores above baseline values at 180 days post treatment were censored. All results were analyzed with IBM SPSS Statistics for Windows, Version 20.0. (Armonk, NY: IBM Corp., released 2011) and a significance level of p < 0.05 was set.

## Data Availability

All data generated or analysed during this study are included in this published article.
